# Delayed Post-Operative Subcutaneous Emphysema

**DOI:** 10.7759/cureus.13051

**Published:** 2021-02-01

**Authors:** Yuchen Jiang, Rajini Rajagopal, Stephanie Germain, Priyamal Silva

**Affiliations:** 1 Otolaryngology, John Radcliffe Hospital, Oxford University Hospitals NHS Foundation Trust, Oxford, GBR; 2 Otolaryngology - Head and Neck Surgery, Oxford University Hospitals NHS Foundation Trust, Oxford, GBR

**Keywords:** aerodigiestive tract, surgical emphysema, subcutaneous emphysema, hypopharynx, endotracheal intubation

## Abstract

The authors present the case of an 87-year-old woman who developed a delayed onset of subcutaneous emphysema post-operatively. We discuss the causative factors - in this case, presumed injury to her hypo-pharynx during a reportedly uneventful endotracheal intubation, the investigations and the management of this rare complication.

## Introduction

Surgical emphysema is a rare complication of endotracheal intubation [1]. In such cases, known causes include trauma from instrumentation during intubation, overinflation of the endotracheal tube cuff or insertion of a feeding tube [2]. Although usually presenting immediately, late-onset surgical emphysema may be due to an increase in alveolar pressure exacerbating an existing injury in the aerodigestive tract. Management is conservative or surgical.

## Case presentation

An 87-year-old lady was admitted under trauma and orthopaedics with a left femur following a fall. Her medical history included osteoporosis, migraine, diverticular disease, polypectomy for malignant neoplasm of colon and previous thyroidectomy. She was otherwise independent with activities of daily living and lived alone. Her femur fracture was managed surgically by fixation with a long gamma nail under general anaesthetic.

During induction of anaesthetic, a consultant anaesthetist used 70mg propofol and 50mg atracurium. It was recorded that whilst the view was grade 3, intubation was uncomplicated. Bougie insertion was straightforward with no holdup and a size 7 endotracheal tube was inserted directly into the trachea. Tracheal clicks were felt and the patient was intubated without significant difficulty.

Intraoperative ventilation was unremarkable with maintenance on sevoflurane. The patient had a nasal temperature probe inserted without resistance. She was extubated by a different consultant anaesthetist without the need for ventilation with a breathing (Water’s) circuit or bag valve mask. Post extubation the patient remained stable and was brought to recovery, where it was noted she had some blood in her mouth. 

**Figure 1:**
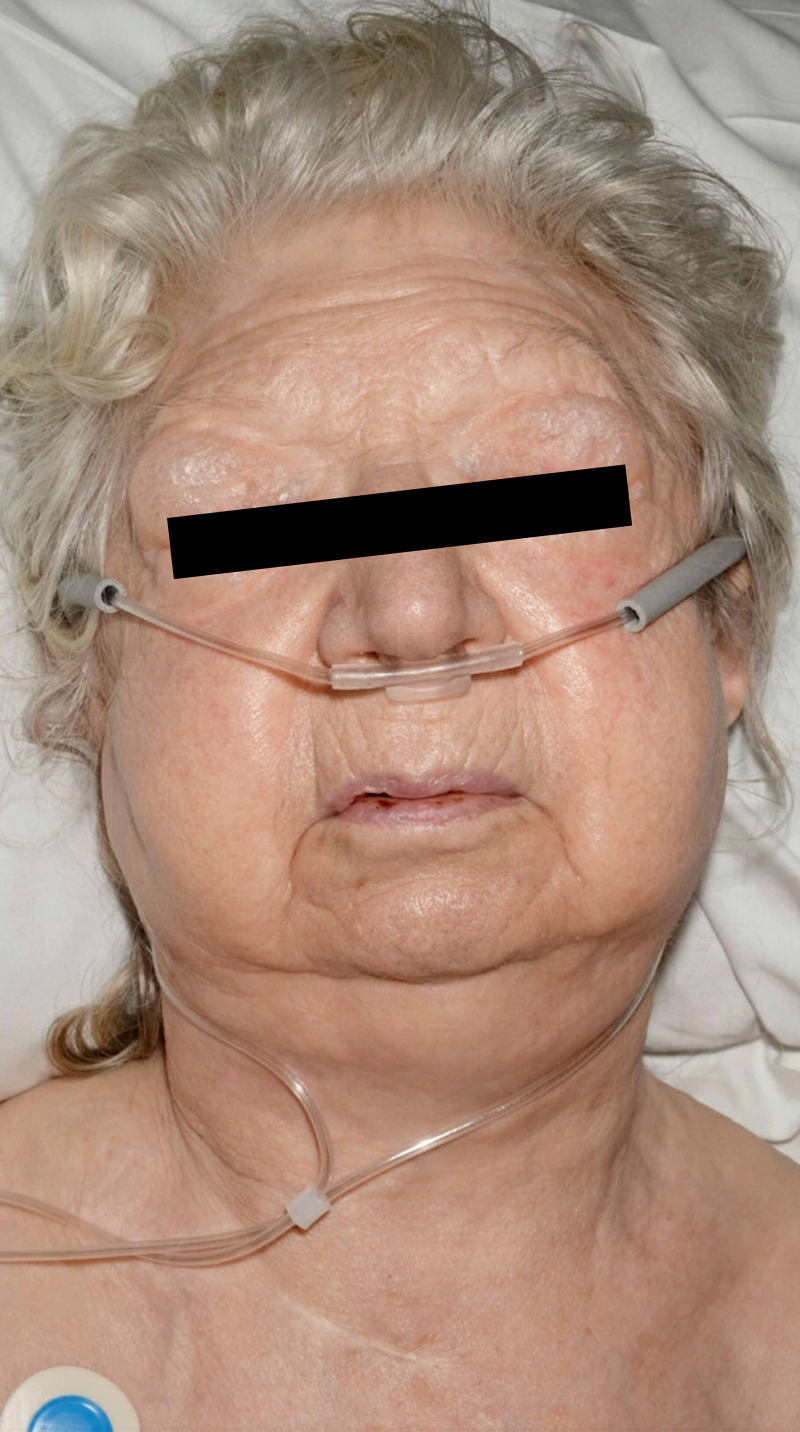
Surgical emphysema over the eyes, cheeks and neck.

**Figure 2:**
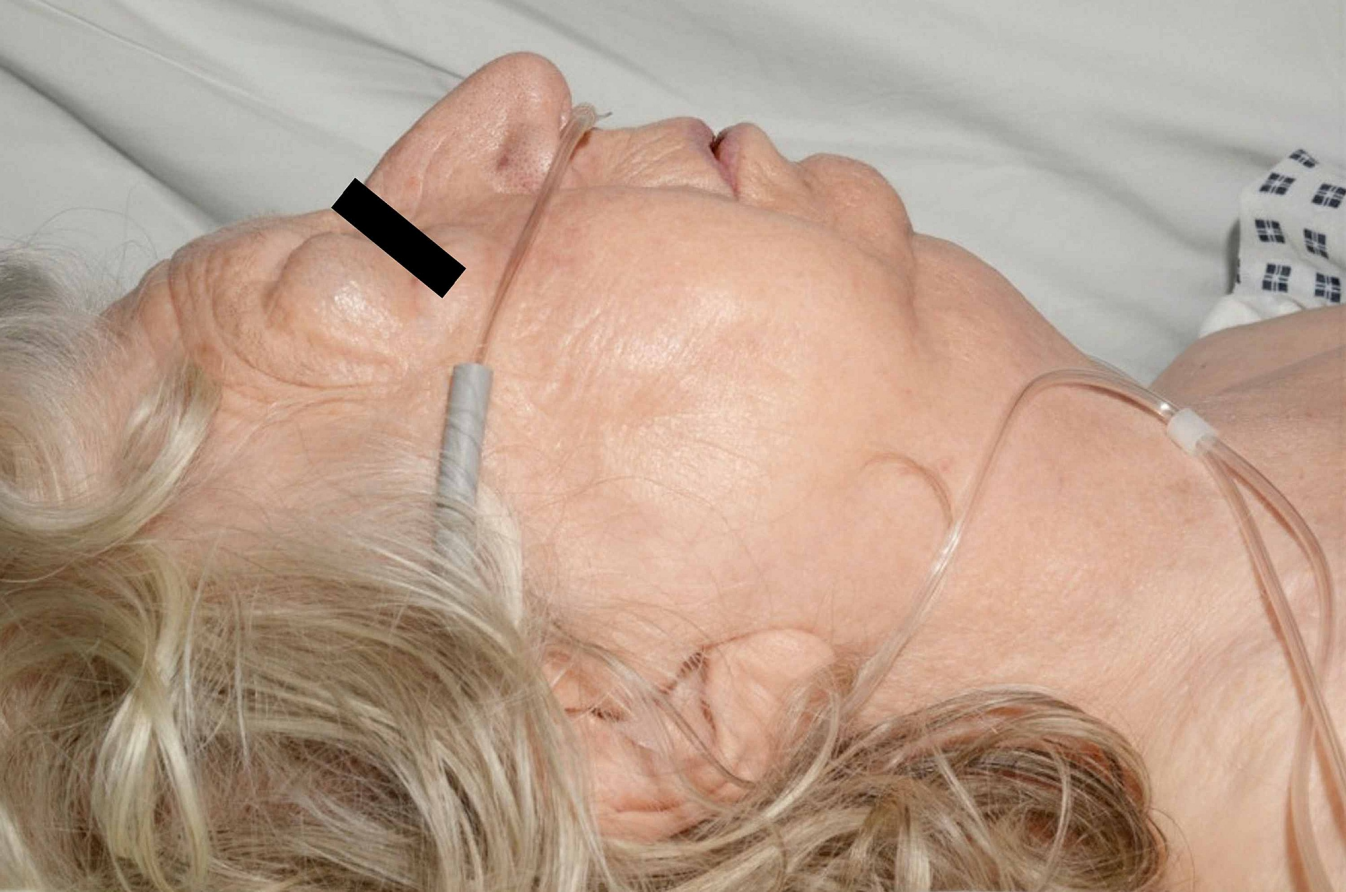
Lateral view of face showing areas of surgical emphysema.

In recovery, after an uneventful first hour, the on-call anaesthetic registrar was alerted to review sudden onset periorbital oedema and swelling of the patient’s face, neck, and chest down to the sternum. (Figures 1, 2) She was able to speak with no lip or tongue swelling and denied pleuritic chest pain. She maintained oxygen saturations of 100% on 15 L of oxygen. There was crepitus on palpation of her face, neck and chest. The impression was that of surgical emphysema and there was no worry of anaphylaxis or allergy.

**Figure 3:**
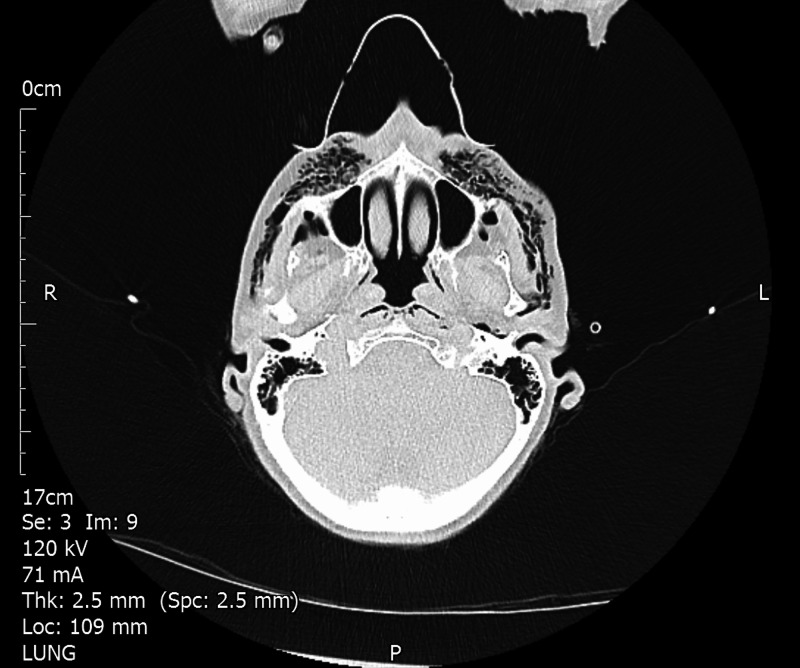
CT showing pockets of subcutaneous overlying the maxilla bilaterally.

An urgent chest X-ray was done which showed extensive surgical emphysema and no signs of a pneumothorax or rib fractures. An urgent computed tomography (CT) scan showed possible injury to the hypopharynx at the level of C2 as well as confirming the surgical emphysema (Figure 3).

Urgent ear, nose, throat (ENT) opinion was sought and examination with a flexible nasal endoscope (FNE) by the consultant ENT surgeon revealed an area of redness and possible trauma in the corresponding area identified on CT. As the patient had no airway compromise the advised management was conservative measures with close monitoring on the adult intensive care unit (ICU) and early intubation if any signs of airway compromise.

Day 1 post-op the patient was trialed on sips of fluids which was initially tolerated. Later that day she became drowsy and developed difficulty swallowing with pooling of saliva. Prior to nasogastric tube insertion, ENT surgeons scoped the patient again. Repeat FNE showed a small tear on the posterior wall of the oropharynx, a small amount of blood in the left piriform fossa, and further injury below the vocal cords could not be ruled out.

The CT images from the previous night were re-discussed with the duty radiologist who was unable to confirm an injury in the hypopharynx or trachea and recommended further investigation with bronchoscopy. The patient was reviewed by the cardiothoracic surgeons who advised continuing conservative treatment as the patient had no airway compromise. A bronchoscopy was therefore not performed.

**Figure 4:**
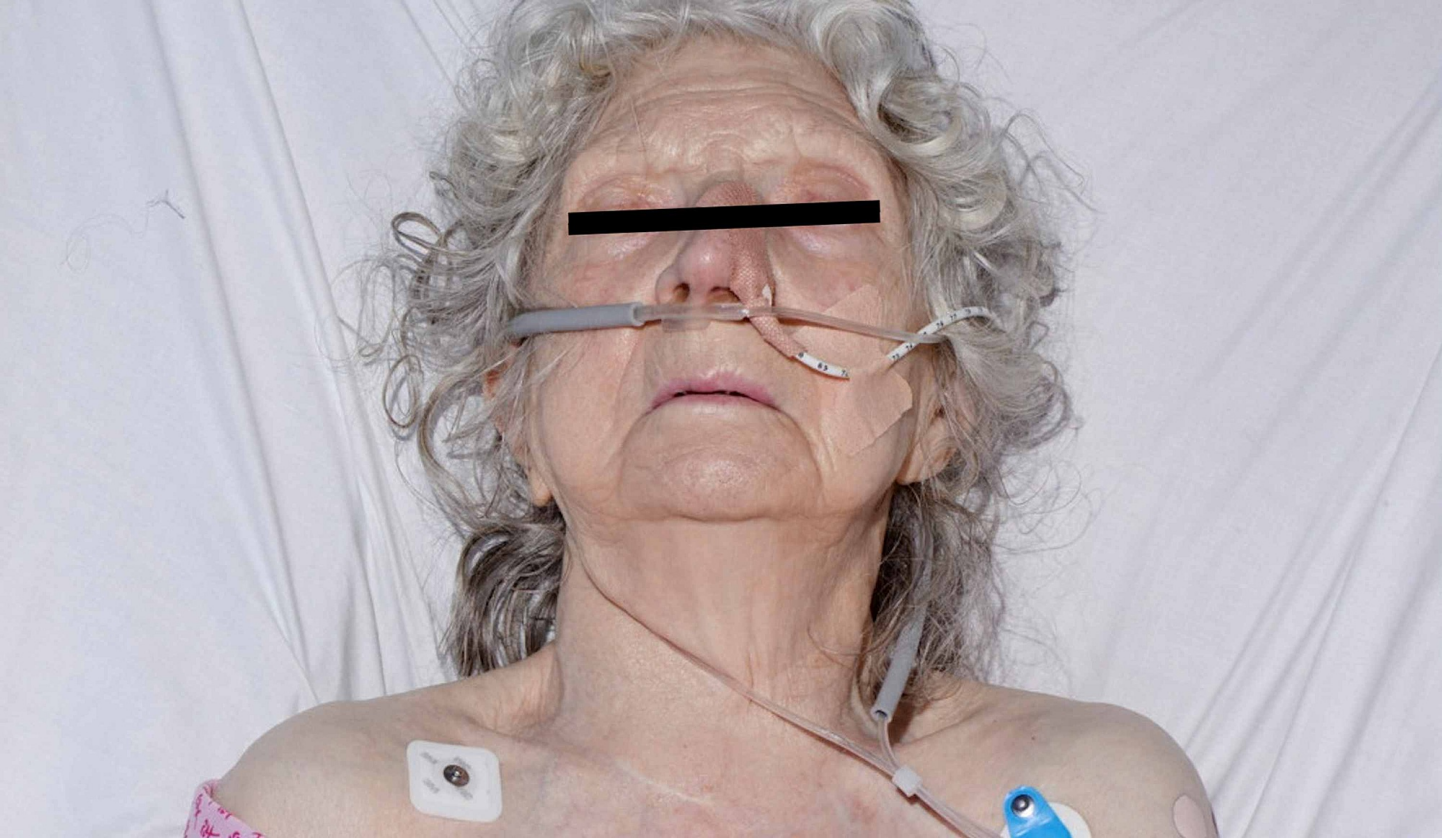
Following resolution of surgical emphysema.

**Figure 5:**
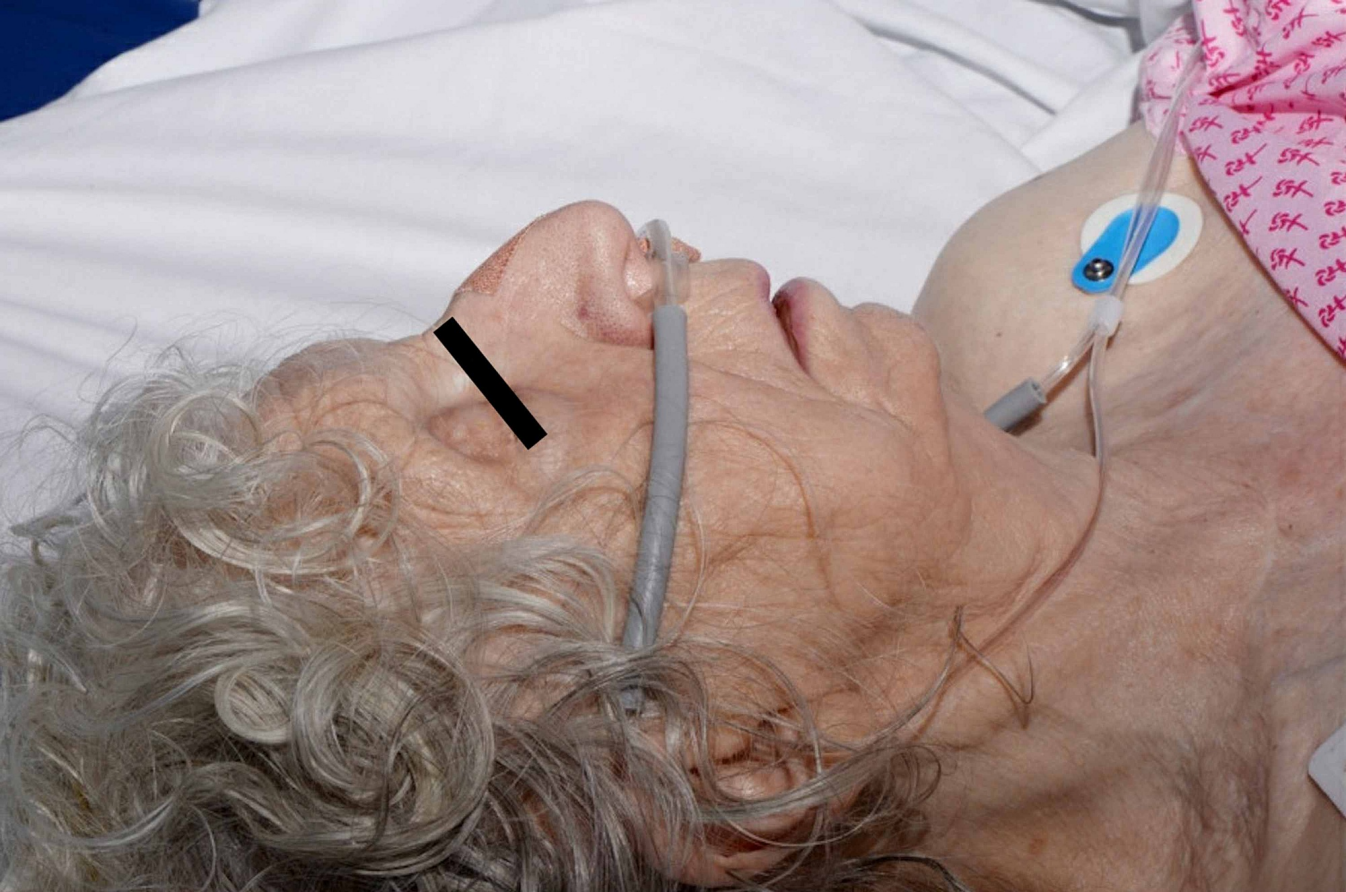
Following resolution of surgical emphysema.

The patient had a fluoroscopically guided insertion of an NG tube for feeding as possible injury below the vocal cords could not be ruled out. She received intravenous co-amoxiclav for seven days before having a barium swallow which ruled out a leak. The NG tube was removed and she was started on a normal diet following input from the speech and language team. Over this time, the surgical emphysema had improved significantly and the patient was stepped down from ICU to an orthopaedic ward for rehabilitation following her initial presentation of fractured femur (Figures 4, 5). The patient was eventually discharged home with a package of care. She suffered no long-term phonation, breathing or swallowing issues and made a full recovery.

## Discussion

Subcutaneous emphysema as a result of hypo-pharyngeal injury is an uncommon but usually immediate complication of endotracheal intubation. There is evidence that perforation of the aero-digestive tract is more likely to occur when thin airway adjuncts such as bougies or stylets are used [3]. An association is seen in patients over the age of 60, in those with friable mucosa due to disease, and in females [4]. There are very few descriptions of delayed post-operative subcutaneous emphysema in the literature. However, it is recognised to occur after vomiting, secondary to raised alveolar pressure from the Valsalva manoeuvre during vomiting [5]. 

In our case, there was no pre-existing pharyngeal disease seen on FNE but it is possible that the patient sustained a very small puncture during intubation, whether from the bougie or ET tube itself. The injury was likely to have been tamponaded by the ET tube in situ during the operation. There is the possibility that an increase in pressures from the patient retching or vomiting post-operatively precipitated the onset of her subcutaneous emphysema.

Imaging with CXR should be done in the first instance to rule out pneumothoraces followed by direct visualisation with FNE. If these are inconclusive, a barium swallow and CT in unwell patients may aid diagnosis and guide surgical management [6].

Given how rare pharyngeal tears occur there is currently no gold standard of treatment. Combined management by the anaesthetist and ENT surgeon is preferable. It is recognised that pharyngoesophageal lesions limited to the pharynx and less than 2 cm may be closely monitored and treated conservatively. Oesophageal lesions are more likely to require surgical repair [7,8].

Where there is concern about breach of the aerodigestive tract, avoidance of perpetuating infection in the deep spaces of the neck (para- and retro-pharyngeal abscess) as well as chest (mediastinitis) is paramount [9]. Principles to avoid these are achieved with fasting the patient orally, with the insertion of NG tubes or central venous access for enteral feeding, and giving broad-spectrum antibiotics. In the event of airway compromise, recommended definite airway is via an awake tracheostomy under a local anaesthetic with surgical exploration of the laryngotracheal trauma to follow [10].

## Conclusions

Cervical subcutaneous emphysema is a rare but potentially life-threatening complication following endotracheal intubation. The onset is normally immediate post-injury but in select instances, presentation may be delayed. Early recognition of clinical signs and prompt escalation of treatment is pivotal to prevent life-threatening complications. Bedside monitoring and investigations followed by selective imaging and direct visualise determine case-by-case management. The use of antibiotics to prevent deep neck space or mediastinum infection from salivary contamination is important to reduce mortality.
